# Identifying and addressing institutional barriers to community partner compensation for engaged research: A Clinical and Translational Science Award case study

**DOI:** 10.1017/cts.2025.79

**Published:** 2025-04-28

**Authors:** Alicia Bilheimer, Nixola Datta, Mary E. Grewe, Simone Frank, Mysha Wynn, Christopher Tunstall, Jubilo Tommy White, Guadalupe C. Hernandez, Lori Carter-Edwards

**Affiliations:** 1 North Carolina Translational and Clinical Sciences Institute, University of North Carolina at Chapel Hill, Chapel Hill, NC, USA; 2 North Carolina Translational and Clinical Sciences Institute, Community and Patient Advisory Board, University of North Carolina at Chapel Hill, Chapel Hill, NC, USA; 3 Kaiser Permanente Bernard J. Tyson School of Medicine, Pasadena, CA, USA

**Keywords:** Community engagement, research financial administration, community partner compensation, institutional transformation, Clinical and Translational Science Award

## Abstract

Little guidance exists for developing institutional policies and procedures that support financial management of community-engaged research, including those related to compensating community partners equitably and efficiently for their expertise and time. To address this gap at our institution, the North Carolina Translational and Clinical Sciences Institute at the University of North Carolina at Chapel Hill (UNC) pursued an iterative, multi-pronged approach to identify and address institutional barriers and facilitators related to community partner compensation for research engagement. This case study describes the approach used to involve research administrative leadership, research teams, and community partners at UNC in the identification of institutional barriers to efficient partner compensation. It also elucidates our efforts to develop policies, processes, and resources to address these barriers. The approaches and solutions described can be adapted by other academic research institutions to enhance compensation processes and to facilitate incorporation of community perspectives into the design and implementation of institutional processes that directly impact their engagement in research.

## Introduction

Community-engaged research (CEnR) is a collaborative approach whereby community members and academic researchers partner throughout the research process, with an emphasis on principles such as co-learning, mutual benefit, and long-term commitment [1]. Over the past several decades, evidence supporting the importance of community engagement in research has grown, as has awareness that community-academic partnerships promote relevant, impactful, and sustainable research [1]. Starting in the mid-2000s, the National Institutes of Health and other sponsors began emphasizing community engagement as a key aspect of their funded research and now substantially invest in the design, implementation, and evaluation of CEnR initiatives [2–4]. The science of engagement, and what makes certain engagement approaches more impactful than others, is nascent [5]. However, it is widely recognized that successful engagement in research requires the creation and maintenance of bidirectional community-academic partnerships where all parties feel trusted, valued, and empowered [6]. Appropriate financial compensation has been linked to community partner trust in research and to perceptions of value placed by academic partners on communities [6]. As such, fair and timely financial compensation to community partners for the dedication of their time and expertise is a crucial aspect of successful engaged research. Financial compensation, however, is influenced by myriad, complex institutional structures and factors beyond the control of academic and community partners themselves.

Federal requirements exist around how and to whom federally sponsored research grants are distributed. Fortunately, many of the fiscal and administrative processes at research institutions that guide community partner compensation are locally dictated and thus more flexible. However, institutional processes are often developed to meet the needs of research and administrative staff, not those of community partners [7]. The Clinical and Translational Science Award (CTSA) Program at the NIH’s National Center for Advancing Translational Sciences requires the institutions it funds to implement programming that promotes engagement of community partners in clinical and translational research [3]. As such, CTSA staff housed within medical research centers are well positioned to integrate community perspectives into institutional processes, advocate for fair financial compensation for community partners, transform institutional structures that impede community engagement, and serve as liaisons between partners, researchers, and institutional leadership [8,9].

The North Carolina Translational and Clinical Sciences (NC TraCS) Institute – the CTSA hub at the University of North Carolina at Chapel Hill (UNC) – has long recognized the importance of identifying and addressing fiscal issues affecting CEnR. In 2013, with the support of NC TraCS, three community partners founded the PRIME Collective, LLC. This organization is led by community experts with extensive experience incorporating principles of community engagement into research and collaborating with universities on the administration of research projects. Given its organizational status, PRIME has a vendor relationship with UNC that allows payments from the university to go directly to the organization, which can then disburse funding to individual community members with whom the organization contracts; through independent community-based infrastructures such as PRIME, academic-community interactions can become more efficient while also enhancing community capacity for fiscal administration. Furthermore, from 2012 to 2015, NC TraCS led conversations with community partners, researchers, and grant administrators at UNC to: 1) identify gaps in skills and knowledge related to navigating pre- and post-award grant periods, and 2) develop comprehensive resource guides for academic researchers and community partners to enhance understanding of the grant submission and management process when conducting federally funded CEnR [10,11].

Expanding upon this prior work, in 2018 NC TraCS led a qualitative research study across four CTSA institutions to identify administrative and fiscal barriers and facilitators to CEnR [12]. Participants included community, academic, and administrative partners affiliated with the four CTSAs. This study revealed challenges inherent in the administrative and fiscal processes across institutions, many of which directly impact the institutions’ abilities to compensate community partners. Most notably, participants described how burdensome institutional infrastructure and policies (including time-consuming financial paperwork and lack of process standardization) can slow fiscal and administrative processes. They highlighted that research administrators may lack experience in and understanding of community partner compensation processes and needs. They also cited that community partners often lack understanding of academic fiscal processes and are burdened by navigating institutional systems not adapted for community organizations. To address these barriers, participants recommended working collaboratively to enhance community partner familiarity with academic policies and processes, promote training and education, share information about institution fiscal practices or requirements, and develop standardized resources [12]. Notably, no best practices were identified related to institutional infrastructure, policies, and procedures that support financial management of CEnR or compensating community partners.

To address this gap at UNC, our CTSA developed and implemented a multi-pronged, community-engaged approach to 1) identify barriers to community partner compensation for research engagement at our university, and 2) develop processes to facilitate the transformation of institutional structures to promote fair and timely compensation of community partners. By using a case study approach, this paper details our process for identifying contextual barriers and developing relevant solutions at our institution. The qualitative research study cited above, as well as ongoing conversations with other CTSAs, suggest that the institutional challenges we face in compensating community partners are not unique, that shared institutional conditions have yielded universal challenges, and that cross-institutional best practices to address these challenges have not been identified. This case study shares practices that may be able to be adapted by others to enhance compensation processes and ensure incorporation of community priorities into the design and implementation of institutional processes that impact research engagement.

## Approach

Starting in 2021, CTSA faculty and staff from the Patient and Community Engagement in Research (PaCER) Program and the Inclusive Science Program at NC TraCS pursued a multi-pronged approach to identify barriers to partner compensation at our university and to develop short- and long-term solutions to address these barriers. CTSA staff gathered input from various sources (including research administrative leadership, research teams, and community partners) to gain a comprehensive understanding of the various factors, including barriers and facilitators, that influence partner compensation.

### Research administrative leadership

In 2021, CTSA faculty and staff initiated conversations with institutional financial administrators (e.g., leadership within the Office of Sponsored Programs and the Accounts Payable and Vendor Services department). These conversations aimed to explore the fiscal challenges inherent in conducting CEnR at UNC, identify barriers to partner compensation, and generate strategies to streamline and improve processes for sub-awards and contracts with community partners. Discussions occurred via email and Zoom and focused primarily on two pain points in the compensation process - the initial set-up of community partners as independent contractors within the institute’s financial administration system, and the timely payment of partners throughout their involvement in a research project. We reviewed dozens of email exchanges and meeting minutes to summarize the institutional barriers and solutions identified, which are described under *Preliminary Outcomes* below.

### Research teams

PaCER serves as a consultative resource for researchers across our university seeking advice related to community engagement. As such, PaCER staff are frequently consulted to help researchers navigate engagement challenges, including those related to community partner compensation. Feedback noted during these consultations, as well as from informal listening sessions with staff and faculty from various research institutes across the university, elucidated the types of daily struggles faced by “boots on the ground” researchers and informed the *Preliminary Outcomes* below.

### Community partners

In 2021, our CTSA established a Community and Patient Advisory Board (CPAB) that provides guidance on CTSA programming and advocates for institutional infrastructure that promotes equitable engagement and participation in research [13]. CPAB members are community leaders, patients, and advocates from across North Carolina with decades of experience as participants and community partners in research [14]. Since the group’s inception, CTSA staff has engaged in group and personal conversations with CPAB members about compensation-related hurdles faced when working with universities. In 2023, CTSA staff led a 2-hour facilitated discussion with four CPAB members to elucidate their experiences with compensation, the challenges they face in receiving payment, and feasible solutions they feel would benefit community partners and research participants.

Similarly, in 2020 our CTSA established a Latine Community Review Board (LCRB) comprised of native Spanish-speaking, Latine North Carolinians who help researchers culturally adapt interventions, improve linguistic accuracy of Spanish language research materials, and enhance participant diversity in clinical research. In 2024, CTSA staff facilitated a 2-hour meeting with eight LCRB members to discuss challenges in payment processing methods specifically for Latine community partners. During discussions with both LCRB and CPAB members, CTSA staff took notes to document what was shared and identify key takeaways.

## Preliminary outcomes

Feedback gathered by our CTSA resulted in our teams’ identification of three pervasive barriers to community partner compensation, as well as solutions for how to address these barriers at UNC. While the barriers we identified, and the solutions we have pursued, are specific to our institutional context, we hope that they are useful for other academic research institutions facing similar structural hindrances to efficient partner compensation.

### Barriers

#### Inaccessibility and poor usability of payment-related forms for community partners

Throughout the communications described above, research teams and community partners cited the inaccessibility and poor usability of payment-related forms as a barrier to partner compensation. Specifically, community partners are often classified as independent contractors (ICs) when working with academic institutions. At our institution, they were required to complete independent contractor checklists (ICCs) along with IRS Form W-9s to receive payment. The content of the ICC is informed by Internal Revenue Service requirements and its purpose is to ensure that the individual providing services is not an employee of the university.

Community partners cited that they rarely receive an explanation from universities or their research partners about the purpose of the ICC and its impact on payment, and that both the title and content of the form felt confusing and overwhelming. They shared that they do not identify as “contractors” or “vendors” and that the questions asked are not well-suited for their roles when engaging in research. Research teams and community partners expressed frustration with the large amount of paperwork required to process payments as small as $50–100, as well as with the requirement that ICCs be updated annually. They cited the need to submit paperwork manually rather than online as an additional hurdle, which limits accessibility for certain populations. In some cases, community partners decided to forgo payment to avoid paperwork burden. The security of private financial information requested in the paperwork, as well as the requirement to provide a social security number or taxpayer identification number, was also noted as an impediment among community partners.

#### Lack of transparency and clarity in institutional payment processes

Lack of transparency and clarity in institutional payment processes was another barrier to partner compensation highlighted in our conversations described above. Specifically, research teams noted that receiving institutional approval to work with their community partners as ICs sometimes take months, and that payment delays decrease community partners’ trust in the research enterprise. They emphasized the need to expedite the processing and payment of community partners (which can sometimes take as long as six months) and suggested the creation of business office-compliant IC invoice templates. Furthermore, because they are often not notified of payment delays, researchers and department staff must track community partner payments as they progress through the university’s various approval processes. Due to the additional time required to track payments, some research teams have considered quarterly, rather than monthly, payments despite their own preferences to pay partners more frequently. Budget reconciliation processes represent an additional challenge at the close of the fiscal year; while not unique to the university setting, these end-of-year pauses in processing new invoices can result in additional 30–60-day delays in partner compensation.

Community partners also shared that disbursement of funds via check can occur two to six months after invoices are submitted. For partners who require access to funds immediately, this delay presents financial challenges and has resulted in some withdrawing their involvement. Community partners from marginalized populations with less access to economic resources may be more reliant on compensation, and thus disproportionately impacted by payment delays. While direct deposit of funds into a bank account helps alleviate delays associated with mailed checks, current enrollment processes pose challenges for some partners. Specifically, these processes require partners to have a bank account, provide a voided check or letter directly from the bank, and provide verbal confirmation of their bank account number.

Lastly, community partners stated that they are not consistently made aware of the tax implications of IC payment, including that they would receive an IRS Form 1099 if they are paid at least $600 in a calendar year from the university, and that payment received from the university is considered taxable income. For some, this can impact their benefits from public assistance programs (e.g., disability benefits, housing assistance, and food assistance programs).

#### Lack of inclusion of community partner perspectives in compensation-related decision-making

Our conversations with community partners and research administrative leadership highlighted that no systematic processes exist for engaging community members in compensation-related decision-making at the university. As such, community perspectives are often not elicited as part of the decision-making processes that impact them. Community partners emphasized the importance of having a “seat at the table” to ensure that compensation processes align with community preferences and needs. They also underscored the importance of considering power dynamics between community partners and university leadership and the need for support when engaging in conversations and decision-making with university leaders.

### Solutions

To address the barriers discussed above, we worked alongside research administrative leadership, research teams, and community partners to adapt and/or develop the following processes and resources. Although the solutions we describe here were specifically designed for implementation within our own institution, other institutions can consider adaptations for use within their own context.

#### Community collaborator form

Conversations between our CTSA and research administrative leadership at our university around the ICC yielded consensus that this form should be revised and adapted for use by community partners. An existing mechanism used to compensate speakers for one-time engagements with the university was identified as a potential model for compensating community partners. This mechanism permitted use of a tailored, lay language form (rather than the standard ICC) and facilitated faster enrollment of partners into the payment processing system. Its use was allowable for individuals earning less than $5,000 annually, which includes most community partners engaging with the university. As such, we worked with financial administrative leadership to develop the *community collaborator form* - a lay language, user friendly adaptation of the ICC. This form is a three-page fillable PDF that can be used specifically by “community collaborators,” defined as “individuals who are paid to review and evaluate a university activity by sharing feedback, suggestions, insights, and concerns based on their perspectives as lay members of communities that may be affected by that University activity.” The form’s content was developed in collaboration with our CTSA’s community partners to ensure relevance and clarity for broad audiences. In 2021, financial administrative staff distributed the form and associated use policies to university researchers via their website, a memo sent to departmental business managers across the university, and general listservs. This form has been used by hundreds of community partners to date, including those who work with our CTSA. In conversations with our team, several community partners and research teams stated that this form has been easier for community partners to complete and has allowed them to submit required financial paperwork more quickly, thus alleviating user burden.

#### Payment process resource documents

Feedback gathered by our team via the approaches described above and echoed in the literature [12,15] highlight the need for resources and education to enhance researcher and community partner understanding of institutional fiscal practices. To supplement toolkits previously developed by our CTSA [10,11], we worked with community partners and financial administrators to develop two documents that clearly outline processes related to community partner compensation. The first resource – intended for research teams – is a multi-page guide for navigating the decisions, considerations, and steps required to enroll community partners in the payment processing system. For each step in the process, it outlines relevant policies, required forms, responsible parties, and ways to address common challenges.

The second resource – intended for community partners – is an informational document aimed at enhancing transparency around institutional processes related to payment. It outlines the community collaborator enrollment process, how compensation requests move through the institutional approval workflow, the reasons behind the timing of payments, and the benefits of enrolling for direct deposit. The document also highlights the impact that compensation can have on public assistance benefits and includes a resource to help calculate taxes due on additional income. CPAB members have reported that this document is helpful in learning the various steps of the compensation process and has empowered them to educate other community groups they work with on these processes.

#### Community memo to institutional research leadership

Key takeaways from the CPAB listening session and LCRB meeting described above informed the content of a formal memo drafted and signed by 13 NC TraCS community partners. This memo was submitted to CTSA leadership and UNC’s Office of the Vice Chancellor for Research (OVCR) in 2023. It aimed to increase awareness among institutional leadership regarding community partners’ and research participants’ compensation-related concerns and further emphasized the institutional barriers that researchers face when engaging communities. In response to the memo, the OVCR hosted discussions with CTSA staff and three community partners to solicit their perspectives on the usability and implementation of a new institute-wide research participant payment platform.

In 2023, the OVCR launched a collaborative effort between the university and healthcare system to increase the synergy, efficiency, and effectiveness of the institution’s clinical research administrative processes. Issues related to CEnR administration that are being addressed as part of this initiative echo those highlighted in the memo, underscoring a promising synergy between university efforts and community needs. Importantly, community engagement leadership from our CTSA currently serves in two working groups affiliated with this effort and with the OVCR’s broader strategic planning process to improve institutional processes affecting CEnR. As these initiatives progress, CTSA staff will have the opportunity to incorporate CPAB members into working group discussions to ensure their perspectives are included in key, institute-wide decision-making.

### Dissemination

The processes and resources described above have been shared with research teams, community partners, research administrative leadership, and other institutions via a variety of platforms. These include: a training series led by our CTSA focused on the basics of research engagement (as of May 2025, 838 researcher and community partner attendees from 40+ institutions); a presentation to a CTSA community engagement special interest group comprised of 170+ members; a panel presentation at the Association for Clinical and Translational Science’s Translational Science 2024 conference in Las Vegas, NV; and our CTSA’s website. Notably, several individuals from other CTSAs have sought our guidance on how to adapt memo-related language and processes to elevate similar issues at their respective institutions. Our CTSA community engagement staff have also integrated referrals to these resources into the consultative guidance that we provide research teams across our institution.

## Discussion

**Figure 1.**
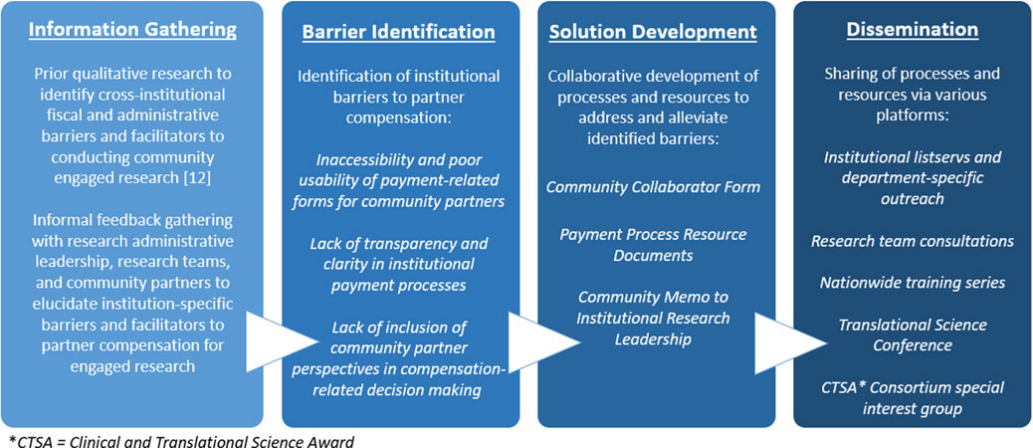
Case study milestones.

Transforming institutional policies and practices to better support community engagement in research is a crucial step toward advancing translational science. However, navigating institutional change is complex, particularly within bureaucratic university settings where institutional administrators (including financial personnel) are often siloed from researchers and community members, and opportunities for information sharing and collaboration are limited [15, 16]. The CTSA is well-positioned to bridge these divides and facilitate knowledge sharing and collaborative change management [8, 9]. Leveraging this position, our CTSA collaborated with research administrative leadership, research teams, and community partners to develop unique solutions to the barriers posed by our institution’s current fiscal and administrative practices (see Fig. 1). First, our community collaborator form addresses challenges inherent in standard independent contractor processes via an institutionally approved payment form that prioritizes ease-of-use and relevancy for community audiences. Furthermore, our payment process resource documents enhance often-lacking transparency related to institutional fiscal processes and timelines. Lastly, our community memo highlights the importance of involving community partners in advocacy efforts to improve the institutional operations and structures that impact them. The uptake of these solutions across our university, and the dissemination of our approaches to other CTSAs, can directly influence how both community partners and research administrative leadership are meaningfully involved in addressing compensation-related issues in CEnR.

Our work, along with that of other CTSAs [15], demonstrates that incremental change in university policy and practice is possible with the involvement and commitment of key institutional leadership. In our CTSA’s case, early rapport building and partnership development with research administrative leadership allowed for transparent conversations related to barriers, challenges, and frustrations. It also facilitated the collaborative identification of ways to improve processes to benefit not only community partners but also administrative staff. These leaders served as champions for change, and their advocacy was and will continue to be essential to our efforts’ success. The alignment in timing of our efforts with broader research administration-led initiatives to improve CEnR at our institution has also provided a visible platform to advocate for changes to promote more meaningful engagement.

Similarly, our CTSA’s trusted relationships with CPAB and LCRB members allowed us to quickly elicit and incorporate community perspectives into the processes and materials described above. Our staff are well-versed in the nuances of community partner compensation and thus were able to process partner payments quickly. However, many community members partner with teams who are less familiar with payment processes. To more effectively support our communities to serve in research advisement and co-leadership roles, we must ensure that all research institutions, research teams, and community partners have a strong foundational understanding of how to navigate fiscal administration processes. Furthermore, community partners with knowledge and experience in research, and in navigating the administrative processes inherent in this type of collaboration, should be provided with opportunities to inform the institutional policies that impact them. By ensuring that these policies and processes reflect their needs and roles, we can improve the efficiency of community-academic engagement.

As researchers work to advance the science of engagement and identify transferrable techniques and processes that lead to effective engagement, case studies that highlight efforts and successes within specific institutional contexts are valuable. Compensation is just one of the many engagement-related processes that could benefit from institutional improvement and standardization; we hope that our CTSA’s efforts described here serve as a framework for how to partner with university leadership and community members to promote institutional transformation that empowers communities to collaborate in research.

### Limitations

The solutions discussed above are in the nascent stages of dissemination and long-term evaluation data on their utility and impact is still lacking. For example, current institutional processes do not facilitate tracking of community collaborator forms separately from ICCs, which limits our ability to gauge user uptake of the new form and its impact on payment processing time. Robust evaluation will require institutional commitment to gathering metrics, as well as our CTSA’s commitment to gathering qualitative feedback from users to better understand how our efforts have influenced CEnR.

### A call to action

Implicit in successful CEnR approaches is the understanding that we must balance evidence and ethics – we must do not only what works, but what we know is right, fair, and equitable [17]. Paying community partners for their time and expertise in efficient, sustainable, and just ways is the obligation of every researcher and institution. Our CTSA’s community partners and staff are steadfast advocates of equitable partner compensation and, as such, we pose the recommendations outlined in Table 1 to our institute and others who are seeking to improve their processes. These recommendations are informed by the conversations described above as well as by our own experiences and knowledge and include solutions we have already implemented as well as others that we plan to implement as our work in this space advances.

**Table 1.** Proposed recommendations to improve community partner compensation processes

	Proposed Recommendations
**Institutional / Systemic Level**	Involve university leadership in efforts to improve administrative and fiscal processes related to community engaged research and alleviate modifiable barriers to compensation
	Systematically involve community members in decision-making processes related to compensation for research participants and community collaborators; ensure opportunities for community members to provide commentary on the institutional policies and processes with which they engage.
	Implement a national standard for community partner pay rates that considers regional differences in cost of living and ensures consistent and fair pay for partners engaging across multiple institutions/projects
	Allow for use of timelier and more accessible methods of disbursing compensation (e.g., electronic cash transfers) and use of other compensation mechanisms (e.g., vouchers or grants) to reduce processing times, paperwork, and requirements associated with taxable income.
	Ensure that institutional policies and practices are tailored to the needs of their users, accurately reflect community research roles, and minimize burden on individuals external to the university.
	Enhance language access and broader accessibility to payment processes and forms (e.g., ensure payment-related forms are compliant with federal plain language guidelines [18], are accurately translated, and are available in languages [19] and accessible file formats [20] used by local communities)
	Create funding mechanisms to support partner compensation for involvement in pre-award engagement efforts
	Develop and disseminate tailored educational resources for study teams and community partners to enhance transparency around payment-related processes and requirements of home institutions
**Research Team/ Project Level**	Institute hourly compensation rates for community partners that accurately reflect the depth and value of their expertise (e.g., pay them the same hourly rate that is charged for staff time on contracted projects)
	Share details about compensation methods and timelines in early conversations with community partners and in agreement and onboarding documents

While our CTSA is advocating for these recommendations and working towards transforming our institution’s approach toward community partner compensation, the circumstances that our community partners and research teams face are not unique to our institution or state. Investing time and resources towards collaboratively identifying and addressing long-standing structures that pose barriers to community engagement is critical toward enhancing the trustworthiness of research and our research institutions.
